# Differential gene expression in the contralateral hemisphere of the rat brain after focal ischemia

**DOI:** 10.1038/s41598-023-27663-8

**Published:** 2023-01-11

**Authors:** Ivan B. Filippenkov, Julia A. Remizova, Alina E. Denisova, Vasily V. Stavchansky, Ksenia D. Golovina, Leonid V. Gubsky, Svetlana A. Limborska, Lyudmila V. Dergunova

**Affiliations:** 1 Institute of Molecular Genetics of National Research Center “Kurchatov Institute”, Kurchatov Sq. 2, 123182 Moscow, Russia; 2 Department of Neurology, Neurosurgery and Medical Genetics, Pirogov Russian National Research Medical University, Ostrovitianov Str. 1, 117997 Moscow, Russia; 3 Federal Center for the Brain and Neurotechnologies, Federal Biomedical Agency, Ostrovitianov Str. 1, Building 10, 117997 Moscow, Russia

**Keywords:** RNA, Neurological disorders, Gene regulatory networks

## Abstract

Ischemic stroke is one of the most severe polygenic brain diseases. Here, we performed further functional genetic analysis of the processes occurring in the contralateral hemisphere (CH) after ischemia–reperfusion injury in rat brain. Comparison of RNA sequencing data for subcortical samples from the ipsilateral hemisphere (IH) and CH after 90 min of transient middle cerebral artery occlusion (tMCAO) and corresponding sham-operated (SO) controls showed four groups of genes that were associated with ischemic processes in rat brain at 24 h after tMCAO. Among them, 2672 genes were differentially expressed genes (DEGs) for IH but non-DEGs for CH, 34 genes were DEGs for CH but non-DEGs for IH, and 114 genes had codirected changes in expression in both hemispheres. The remaining 16 genes exhibited opposite changes at the mRNA level in the two brain hemispheres after tMCAO. These findings suggest that the ischemic process caused by a focal ischemia induces complex bilateral reactions at the transcriptome level in the rat brain. We believe that specific genome responses in the CH and IH may provide a useful model for the study of the potential for brain repair after stroke.

## Introduction

Ischemic stroke is a multifactorial disease with a complex etiology and global consequences^1,2^. Cerebral ischemia causes a cascade of biochemical changes in brain tissues^3^. During cerebral ischemia, the induction of glutamate-mediated pathways triggers cell death by massive calcium influx following vessel occlusion^4,5^, and the lack of oxygen causes failure of the respiratory chain and mitochondrial function^6,7^. These changes lead to an inflammatory reaction, which is accompanied by an acute imbalance of matrix metallopeptidases^8^. Violation of the function of the blood–brain barrier (BBB) allows the invasion of immune cells and pathogenic factors into brain cells^9,10^. Similar effects caused by disturbance of the BBB, such as accumulation of excess oxygen radicals and activation of apoptosis, can also be caused by reperfusion after ischemia^11,12^. The involvement of these processes in the development of ischemic injury can be observed at the transcriptome and proteome levels using stroke models in small laboratory animals^3,13–21^.

Ischemia-related metabolic changes occur in both the damaged ipsilateral hemisphere (IH) as well as in the opposite contralateral hemisphere (CH) of the brain^22,23^. The processes of transhemispheric diaschisis and spreading depression/depolarization after focal ischemic damage are associated with massive transmembrane ion shifts distant from the original site of injury^23–26^. The gene expression patterns can change in the CH after focal cerebral ischemia^9,27–30^. In experiments involving the CH and sham-operated (SO) controls, we recently found traces of a bilateral genetic response after focal stroke induced by transient middle cerebral artery occlusion (tMCAO) in the rat brain^31^. Comparisons with control conditions identified overlapping differentially expressed genes (DEGs) that reflected the general transcriptome response of IH subcortical cells at 24 h after tMCAO. We also identified sets of nonoverlapping genes that are unique in the SO and CH controls. These DEGs may indicate that transcriptome changes in IH subcortical cells are associated with transhemispheric differences in models of ischemia. The functional annotation of gene sets reveals an association with inflammatory and immune reactions occurring in the CH zone in response to focal ischemia–reperfusion (IR) injury^31^. However, the role of genomic parameters in the CH in the mechanisms of ischemic injury and neuroprotection remains underestimated and requires further study.

In the present study, we performed further functional genetic analysis of the processes occurring in the CH after IR injury. Comparison of RNA sequencing (RNA-Seq) data for rat brain subcortical samples from the IH and CH after 90 min of tMCAO and corresponding SO controls showed four groups of genes that were associated with ischemic processes in rat brain at 24 h after tMCAO. Among them, 2672 genes were DEGs for IH but non-DEGs for CH, 34 genes were DEGs for CH but non-DEGs for IH, and 114 genes had codirected changes in expression in both hemispheres. The remaining 16 genes (*Hrh3*, *Chst15*, *Drd2*, *Rasd2*, *Drd1*, *Hpca*, *Lrrc10b*, *Slc24a4*, *Scn4b*, *Neu2*, *Gng7*, *Adora2a*, *Asic4*, *Syndig1l*, *Rgs9*, and *Gpr6*) exhibited opposite changes at the mRNA level in the two brain hemispheres after tMCAO. Interestingly, the last gene group comprising 16 DEGs was associated predominantly with the functioning of the neurosignaling system. These findings suggest that the ischemic process caused by a focal ischemia induces complex bilateral reactions at the transcriptome level in the rat brain. We believe that specific genome responses in the CH and IH may provide a useful model for the study of the potential for brain repair after stroke.

## Results

### RNA-Seq analysis of the effects of IR on the mRNA level in subcortical structures of the CH relative to the SO controls

**Figure 1:**
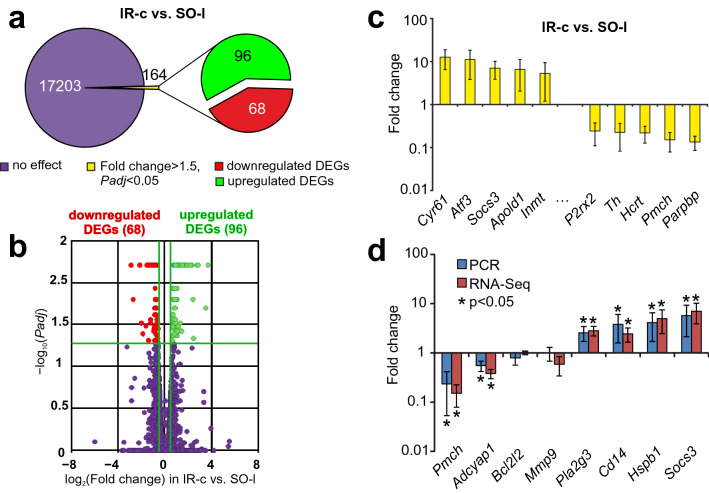
RNA-Seq analysis of DEGs at 24 h after tMCAO in subcortical structures of the CH related to the corresponding brain samples from SO rats. (**a**) RNA-Seq results for IR-c versus SO-l. The numbers in the diagram sectors indicate the number of DEGs. (**b**) A volcano plot shows a comparison of the distribution of genes between the IR-c and SO-l groups. Upregulated and downregulated DEGs are represented as red and green dots, respectively (fold change > 1.50. *Padj* < 0.05). Not differentially expressed genes (non-DEGs) are represented as dark purple dots (fold change ≤ 1.50. *Padj* ≥ 0.05). (**c**) The top 10 genes that exhibited the greatest fold change in expression in IR-c vs. SO-l. The data are presented as the mean ± standard error (SE) of the mean. (**d**) RT–PCR verification of the RNA-Seq results. Data for the comparison between IR-c and SO-l are shown. Two reference mRNAs *Gapdh* and *Rpl3* were used to normalize the PCR results. Each group included at least five rats. Six genes whose expression changed by > 1.5-fold from the baseline value and whose *P*-value was < 0.05 and two other genes were selected for analysis. The data are presented as the mean ± SE.

We previously used RNA-Seq to evaluate the transcriptional activity of the mRNAs for 17,367 genes in the subcortical structures of the rat brain at 24 h after tMCAO. A pairwise comparison for the primary analysis of the RNA-Seq data for IR-c vs. SO-l revealed significant changes at the mRNA level for 164 genes at 24 h after tMCAO. Here, we analyzed these DEGs further and found that the expression levels of most of these DEGs were higher in the IR-c than in the SO-l group (96 upregulated vs. 68 downregulated DEGs) (Fig. 1a, see Supplementary Table S1). The volcano plot in Fig. 1b shows the differences in mRNA expression levels between the IR-c and SO-l groups. We note that upregulated DEGs included *Cyr61*, *Atf3*, *Socs3*, *Apold1*, and *Inmt*, which were upregulated by ≥ 5 times. The top five downregulated DEGs in IR-c vs. SO-l were *P2rx2*, *Th*, *Hcrt*, *Pmch*, and *Parpbp*, in which the mRNA level was reduced by ≥ 4 times (Fig. 1c).

RT–PCR analysis of the expression of four upregulated (*Pla2g3*, *Cd14*, *Hspb1*, *Socs3*), two downregulated (*Pmch*, *Adcyap1*), and two nonsignificantly (*Bcl2l2*, *Mmp9*) regulated genes was used to verify the RNA-Seq results in IR-c vs. SO-l. The characterization of the primers is shown in Supplementary Table S2. The real-time RT–PCR results confirmed the RNA-Seq data (Fig. 1d).

### Comparison of RNA-Seq results and identification of specific genome responses in the CH and IH

**Figure 2:**
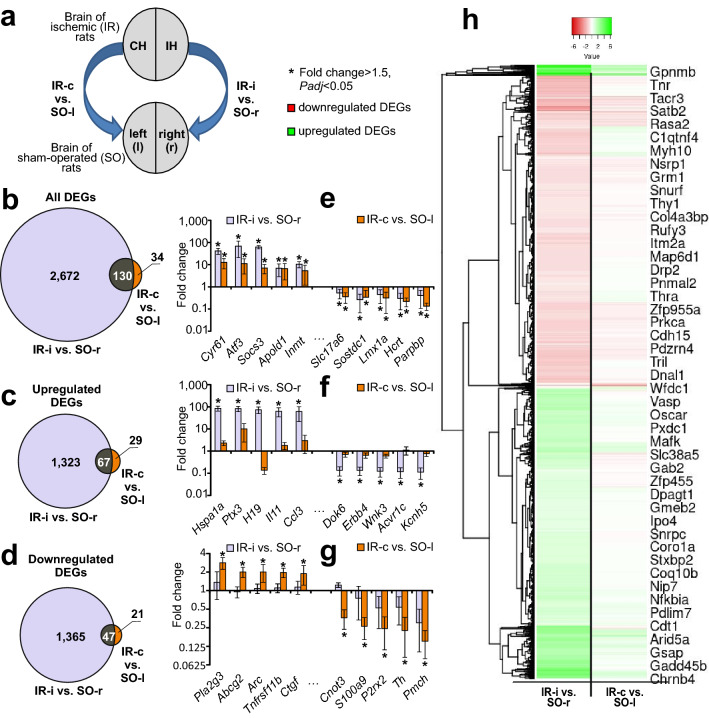
Comparison of RNA-Seq results for the CH and IH relative to the SO controls. (**a**) Comparisons of data are presented. Blue arrows indicate comparisons of ischemic (IR) brain samples versus the respective hemisphere in SO control rats. (**b**–**d**) Schematic comparisons of the results obtained for pairwise comparisons of IR-i vs. SO-r and IR-c vs. SO-l as represented by Venn diagrams. Comparison for all (**b**), upregulated (**c**), and downregulated (**d**) DEGs. The cutoff for gene expression changes was 1.50-fold, and only those genes with *Padj* < 0.05 were selected for analysis. (**e**–**g**) The top 10 genes that exhibited the greatest fold change in expression for IR-i vs. SO-r (**f**) or IR-c vs. SO-l (**e**, **g**) and that were within the gene sets on the Venn diagram are presented (**b**–**d**). DEGs that overlapped in pairwise comparisons of IR-i vs. SO-r and IR-c vs. SO-l are shown (**e**). DEGs for IR-i vs. SO-r but non-DEGs for IR-c vs. SO-l are shown (**f**). DEGs for IR-c vs. SO-l but non-DEGs for IR-i vs. SO-r are shown (**g**). Data are presented as the mean ± SE. Genes whose fold change was > 1.50 and *Padj* < 0.05 relative to the comparison group are marked with an asterisk (*). (**h**) Hierarchical cluster analysis of all DEGs for IR-i vs. SO-r and IR-c vs. SO-l. Each column represents a comparison group, and each row represents a DEG. Green stripes represent a high relative expression level and red stripes represent a low relative expression (n = 3 per group).

Using RNA-Seq, we previously identified 2802 DEGs with 1390 upregulated and 1412 downregulated mRNAs in the rat ipsilateral subcortex relative to the corresponding brain samples from SO rats (IR-i vs. SO-r)^31^. Here, we performed a meta-analysis of the mRNA sequencing results. We analyzed the effects of focal IR on the mRNA levels in each of the hemispheres of the brain and compared these to the levels in the corresponding SO control (Fig. 2a). The results of the pairwise comparisons of the data for IR-i vs. SO-r and IR-c vs. SO-l are presented in the Venn diagram in Fig. 2b. The number of DEGs was 10 times lower in the CH (IR-c vs. SO-l) than in the IH (IR-i vs. SO-r) groups. Simultaneously, we found 130 overlapping genes whose expression changed in both hemispheres at 24 h after tMCAO (Fig. 2b). The Venn diagrams shown in Fig. 2c include only the upregulated DEGs and those shown in Fig. 2d show only the downregulated DEGs for both comparisons.

We identified 67 DEGs that were upregulated in both the comparisons for IR-i vs. SO-r and IR-c vs. SO-l (Fig. 2c) and 47 DEGs whose mRNA was downregulated in both pairwise comparisons (Fig. 2d). Thus, the expression level of 114 of 130 overlapping DEGs between IR-i vs. SO-r and IR-c vs. SO-l changed codirectionally in both hemispheres at 24 h after focal IR. The top 10 overlapping genes with the greatest fold change between the IR-c vs. SO-l groups are presented in Fig. 2e. Among them were genes encoding proteins with transcription factor (*Atf3*, *Lmx1a*), catalytic (*Socs3*, *Inmt*), lipid and protein binding (*Apold1*, *Sostdc1*), and transporter (*Slc17a6*) activities based on the PANTHER data. A full list of these DEGs is included in Supplementary Table S3.

The Venn diagram in Fig. 2e shows 2672 and 34 DEGs that were unique for the effects of IR in the IH (IR-i vs. SO-r) and CH (IR-c vs. SO-l) transcriptomes, respectively. Thus, among the 2672 genes unique to the comparison between IR-i and SO-r were genes encoding proteins of the neurotransmission system (*Gria3*, *Adcy1*, *Grm5*, *Neurod6*, *Kcna2*, and *Kcnh5*); components of the immune system (*Ccl3*, *Ccl7*, and *Ccl22*); the 70-kD heat shock protein (*Hspa1a*), nitric oxide synthase (*Nos3*); and others (see Supplementary Table S4). Among the 34 DEGs unique to the comparison between IR-c and SO-l were *Pla2g3*, *Htra1*, *Tnfrsf11b*, *Iqgap3*, *S100a9*, *Avp*, *Nr4a3*, *Folr1*, *Pmch*, and other genes encoding transcription factors, hormones, and immune response proteins (see Supplementary Table S5). The differential expression data for the top five unique genes for the comparisons IR-i vs. SO-r and IR-c vs. SO-l flanks (relative complements) of the Venn diagram (Fig. 2a) are presented in Fig. 2f and g, respectively.

Hierarchical cluster analysis of all DEGs for the comparisons IR-i vs. SO-r and IR-c vs. SO-c is illustrated in Fig. 2h. The common features of the differential expression profiles of these comparison groups reflect the effects of IR in both rat brain hemispheres. At the same time, individual differences between the groups seem to characterize the specific genome responses in the CH and IH at the transcriptome level.

### Opposite directionality of gene expression changes induced by focal IR in the two rat brain hemispheres at 24 h after tMCAO

**Figure 3:**
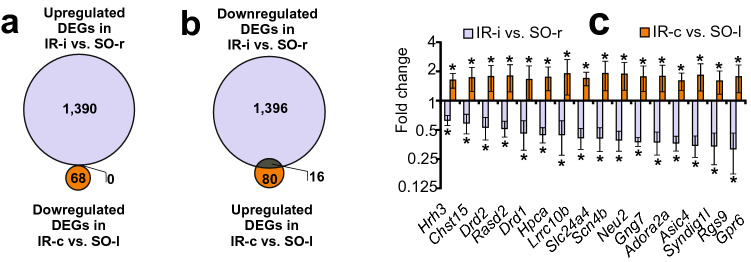
Comparison of RNA-Seq results in the CH and IH relative to the SO rats showed an opposite directionality of changes in gene expression in two rat brain hemispheres at 24 after tMCAO. (**a**, **b)** Schematic comparisons of the results obtained in pairwise comparisons for IR-i vs. SO-r and IR-c vs. SO-l are represented by Venn diagrams. Comparison of only the upregulated DEGs for IR-i vs. SO-r and downregulated DEGs for IR-c vs. SO-l (**a**), and downregulated DEGs for IR-i vs. SO-r and upregulated DEGs for IR-c vs. SO-l (**b**) are shown. The cutoff for gene expression changes was 1.50-fold. Only those genes with *Padj* < 0.05 were selected for analysis. **C**. Sixteen DEGs that were downregulated for IR-i vs. SO-r and, conversely, upregulated for IR-c vs. SO-c are presented and were shown to lie within the intersection of the gene sets on the Venn diagram (**b**). Data are presented as the mean ± SE.

Venn diagrams for only the upregulated DEGs for IR-i vs. SO-r and only the downregulated DEGs for IR-c vs. SO-l are shown in Fig. 3a. Those for only downregulated DEGs for IR-i vs. SO-r and only upregulated DEGs for IR-c vs. SO-l are shown in Fig. 3b. There were no DEGs in the first comparison (Fig. 2c) but 16 DEGs in the second comparison (Fig. 2d): *Hrh3*, *Chst15*, *Drd2*, *Rasd2*, *Drd1*, *Hpca*, *Lrrc10b*, *Slc24a4*, *Scn4b*, *Neu2*, *Gng7*, *Adora2a*, *Asic4*, *Syndig1l*, *Rgs9*, and *Gpr6* were identified. Each of these was downregulated for IR-i vs. SO-r and, conversely, upregulated for IR-c vs. SO-l. These results suggest an opposite directionality of the changes in expression for these DEGs in the two rat brain hemispheres. The differential expression data for these DEGs for IR-i vs. SO-r and IR-c vs. SO-l are shown in Fig. 3c. Using the PANTHER functional annotation tool, we found that these genes are associated predominantly with the neurosignaling system.

### Signaling pathways associated with DEGs in the CH of the rat brain at 24 h after tMCAO

Using DAVID, we found only one signaling pathway (rno05133:Pertussis) that was associated with DEGs for the comparison IR-c vs. SO-l. The GSEA tool, which provides information about enrichment using a more gentle statistical approach, revealed > 100 signaling pathways (FDR q-value < 0.05) associated with DEGs for IR-c vs. SO-l. The top five included Orexin receptor, AP1, IL18, IL6, and IL7 pathways, and signaling by receptor tyrosine kinases (see Supplementary Table S6). Most of the signaling pathways, including those listed, were associated predominantly with upregulated DEGs for the comparison IR-c vs. SO-l. Some overlapped between the IR-c vs. SO-l and IR-i vs. SO-r enrichment results. Concomitantly, four signaling pathways unique for the CH were identified that were associated with DEGs (cutoff > 3.5-fold) for IR-c vs. SO-l but not for IR-i vs. SO-r (see Supplementary Table S6). These included p38 signaling mediated by MAPKAP kinases, signaling pathways for the regulation of TLR by endogenous ligand, hypertrophy model, and extracellular vesicles in the crosstalk of cardiac cells. We note that upregulated DEGs were associated predominantly with each of the signaling pathways identified in the pairwise comparisons (see Supplementary Table S6).

### Analysis of the involvement of the DEGs that had codirectionally changed mRNA level in two brain hemispheres after tMCAO in the signaling pathways that were modulated during ischemia

**Figure 4:**
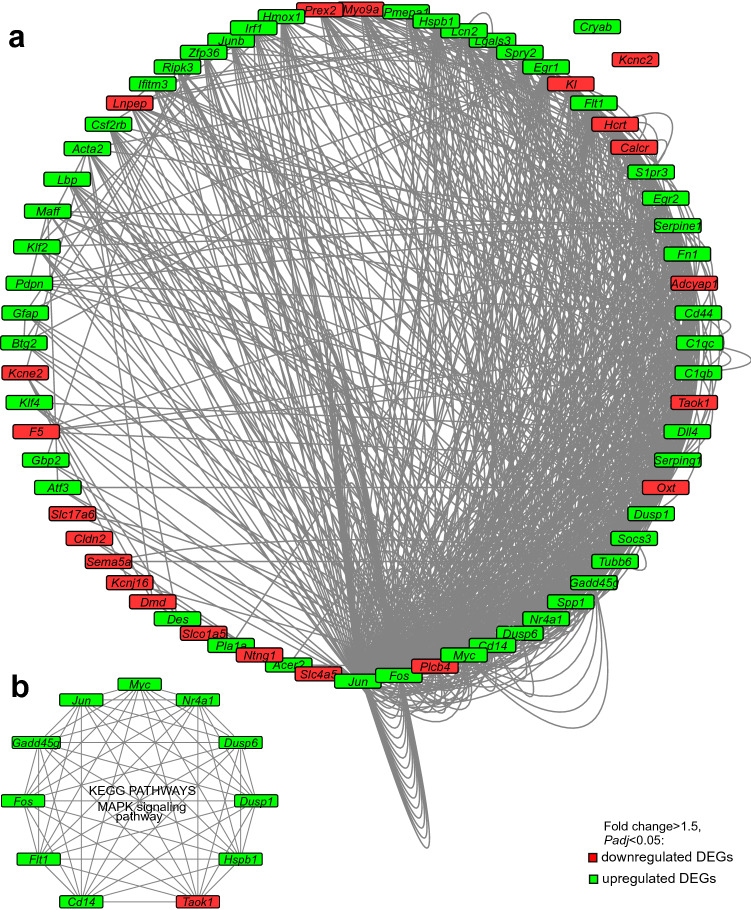
The functional networks of the DEGs with codirectionally changed mRNA levels in the two brain hemispheres after tMCAO. (**a**) In 69 of the 114 DEGs analyzed, significant associations with signaling pathways were observed using DAVID. (**b**) The DEGs associated with the MAPK signaling pathway (KP). Only those DEGs that had codirectionally changed mRNA levels (cutoff > 1.5; *Padj* < 0.05) in the comparisons IR-i vs. SO-r and IR-c vs. SO-l were selected for analysis. Only those pathways associated significantly (*Padj* < 0.05) with DEGs in the comparison IR-i vs. SO-r were selected for analysis. The networks were constructed using Cytoscape 3.8.2 software. The nodes indicate DEGs. Each line connecting the nodes indicates an involvement of the protein product of the corresponding gene in the signaling pathway functioning.

We analyzed 114 DEGs (67 upregulated and 47 downregulated) that had codirectionally changed at the mRNA level in the two brain hemispheres at 24 h after tMCAO (Fig. 2c,d). Using DAVID, the signaling pathway annotations (KEGG PATHWAY (KP), REACTOME PATHWAY (RP), and WIKIPATHWAYS (WP)), were downloaded for these DEGs. Based on previous data^31^, we selected only those annotations with a *Padj* < 0.05 for each set of DEGs for the comparison IR-i vs. SO-r. This produced a list of 133 KP, 21 RP, and 6 WP annotations that were associated with 69 of 114 analyzed DEGs (see Supplementary Table S7). The remaining 45 DEGs analyzed did not have significant annotations identified using DAVID. The association of DEGs with functional annotations showed the participation of 48 upregulated (e.g. *Jun*, *Fos*, *Hspb1*, and *Cd14*) and 21 downregulated (e.g. *Plcb4*, *Oxt*, *Taok1*, and *Adcyap1*) DEGs in the signaling pathways that exhibited codirectional changes in their activities under IR conditions in the subcortical structures of both hemispheres of the brain at 24 h after tMCAO (Fig. 4A).

In the network presented in Fig. 4, nodes are designated as DEGs, and each line connecting the nodes indicates an involvement of the protein product of the corresponding gene in signaling pathway functioning. All genes except for *Cryap* and *Kcnc2* had connections with the other genes on the list analyzed (Fig. 4a). The lines indicate joint involvement of those genes in signaling pathway functioning. In the network (Fig. 4a), the top five genes involved in the most signaling pathways were *Plcb4* (57 pathways), *Jun* (50 pathways), Fos (44 pathways), *Myc* (32 pathways), and *Gadd45g* (19 pathways). The signaling pathways “Signal transduction” (RP), “Immune system” (RP), and “MAPK signaling pathway” (KP) were associated with the most DEGs on our list: 20, 14, and 11 DEGs, respectively.

We note that significant associations of DEGs with the MAPK signaling pathway were revealed by both the KP and WP annotation. The network showing the involvement of the DEGs analyzed in the MAPK signaling pathway (KP) is shown in Fig. 4b. Predominantly, upregulated DEGs were associated with such pathway. Among them were *Cd14*, *Flt1*, *Fos*, *Gadd45g*, *Jun*, *Myc*, *Nr4a1*, *Hspb1*, *Dusp1*, and *Dusp6*. Only one gene (*Taok1*) that is associated with the MAPK signaling pathway was downregulated in both the CH and IH relative to the SO controls at 24 h after tMCAO (Fig. 4b).

The networks shown in Fig. 4 show the spectrum of genes and their functional connections that reflect changes in the activities between different hemispheres of the brain at 24 after tMCAO. Such genes are associated predominantly with modulation of the immune and inflammatory responses in rat brain cells under IR conditions.

### Analysis of the involvement of the DEGs that exhibited opposite changes in the mRNA level in two brain hemispheres after tMCAO in the signaling pathways modulated during ischemia

**Figure 5:**
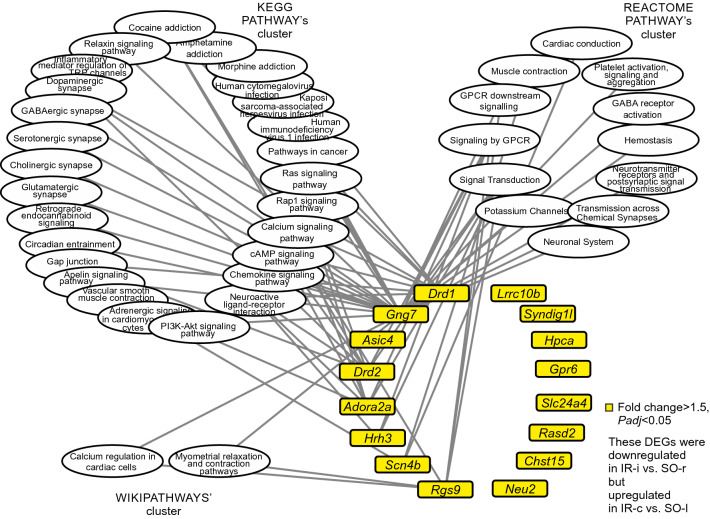
Network showing the involvement of the DEGs that had oppositely changed mRNA levels for the signaling pathways modulated during IR in the two brain hemispheres after tMCAO. The network was constructed using Cytoscape 3.8.2 software. The nodes are designated as the DEGs or signaling pathways. Each line connecting the nodes indicates the involvement of the protein product of the corresponding gene in signaling pathway functioning. All clustered signaling pathways were annotated using KP, RP, and WP databases. The cutoff for mRNA expression changes was 1.50. Only those DEGs and annotations with *Padj* < 0.05 for each set of DEGs in the comparison IR-i vs. SO-r were selected for analysis.

Next, we analyzed 16 DEGs (*Hrh3*, *Chst15*, *Drd2*, *Rasd2*, *Drd1*, *Hpca*, *Lrrc10b*, *Slc24a4*, *Scn4b*, *Neu2*, *Gng7*, *Adora2a*, *Asic4*, *Syndig1l*, *Rgs9*, and *Gpr6*) that were downregulated in IR-i vs. SO-r but upregulated in IR-c vs. SO-l (Fig. 3c). Similarly, based on previous data^31^, only those annotations (KP, RP, WP) with a *Padj* < 0.05 for the set of DEGs in the comparison IR-i vs. SO-r were selected for analysis. We formed a list of 27 KP, 12 RP, and 2 WP annotations that were associated with eight of the 16 DEGs analyzed. The remaining four DEGs analyzed did not have significant annotations identified using DAVID. The association of DEGs with functional annotations suggest the participation of *Hrh3*, *Drd2*, *Drd1*, *Scn4b*, *Gng7*, *Adora2a*, *Asic4*, and *Rgs9* in the signaling pathways that exhibited changes in their activities under IR conditions in the subcortical structures of the brain at 24 h after tMCAO (Fig. 5).

In the network shown in Fig. 5, nodes are designated as DEGs or signaling pathways. Each line connecting the nodes indicates the involvement of the protein product of the corresponding gene in signaling pathway functioning. All signaling pathways were clustered and annotated using the KP, RP, and WP databases. In the network shown in Fig. 5, the top five genes involved in the most signaling pathways were *Gng7* (29 pathways), *Drd1* (eight pathways), *Drd2* (eight pathways), *Adora2a* (seven pathways), and *Rgs9* (six pathways). Two or more of the genes analyzed are involved in the implementation of 13 signaling pathways. The signaling pathways “Signal Transduction” (RP) and “Signaling by GPCR” (RP) were associated with the most DEGs (five) on our list; *Hrh3*, *Drd2*, *Gng7*, *Adora2a*, and *Rgs9* genes were associated with each of these signaling pathways. Additionally, we identified signaling pathways, “Neuroactive ligand-receptor interaction” (KP), “Cocaine addiction” (KP), “cAMP signaling pathway” (KP), and “Dopaminergic synapse” (KP) that were associated with four, three, three, and three DEGs from our list, respectively (Fig. 4). Interestingly, the signaling pathways were associated predominantly with the functioning of the neurosignaling system.

We note that all DEGs analyzed had opposite directionality of expression changes in the two rat brain hemispheres relative to the corresponding SO controls. Thus, this network shows the spectrum of genes and functional-related systems whose activities may change in different hemispheres of the brain at 24 after tMCAO.

## Discussion

In present study, we performed functional genetic analysis of the processes occurring in the CH after a focal stroke. We have previously used the tMCAO (90 min) rat model under MRI monitoring^31^. This model mimics the effects of ischemic stroke events in humans^32,33^. All rats in the IR group had a focal hemispheric lesion lying in the IH region and that spread to the adjacent cortex^31^. We found no pathological changes in the CH of ischemic rats in the MRI data^31^. Based on the RNA-Seq data for this series of rat brain samples from the IH and CH and corresponding SO samples^31^, here, we identified 96 upregulated and 68 downregulated DEGs in the rat CH subcortex. This result suggests the occurrence of latent processes in the CH after stroke. Functional clustering of the gene expression results showed modulation of the Orexin receptor, receptor tyrosine kinases, AP1, IL18, IL6, IL7, and other signaling pathways that are involved in the immune response in CH subcortical cells at 24 h after tMCAO. RT–PCR analysis of the expression of the four upregulated DEGs (*Pla2g3*, *Cd14*, *Hspb1*, and *Socs3*) that are associated with the inflammatory, immune, and stress responses confirmed the RNA-Seq results in the CH. The obtained data support the previously established immunomodulatory effects after ischemia in brain areas away from the focal injury^31,34,35^.

Using RNA-Seq, we previously compared hundreds of DEGs in the rat IH subcortex relative to the corresponding brain samples from SO rats^31^. Here, our comparison of the sets of DEGs in the IH and CH relative to the left and right hemispheres in SO animals, respectively, identified four groups of genes (see Supplementary Fig. S1 online). First, we found genes that overlapped between the pairwise comparisons and that had a codirectional change in expression in both the IH and CH. These genes reflect the general effects of ischemia on the genome in brain cells located at different distances from the stroke site. These genes are associated predominantly with modulation of immune and inflammatory responses (e.g. immune system, MAPK, TNF signaling pathway) in rat brain cells under IR conditions. Thirteen of these DEGs were also identified by Fury et al. using a mouse tMCAO model with 30 min filament period at 1 day after occlusion (brain area + 3.1 to − 4.1 mm from bregma)^30^. *Socs3*, *Inmt*, *Lbp*, *Maff*, *Serpine1*, *Angptl4*, *Gbp2*, *Ifitm3*, *Gpnmb*, *Gfap*, *Emp1*, *Upp1*, and *Acer2К* found in our study and that by Fury et al. were upregulated in both studies and belong to the first group of genes that exhibited a codirectional change in expression in both the IH and CH. Analysis of the results obtained in two pairwise comparisons of IR-c vs. SO-l between Fury et al. and our present studies is shown in Supplementary Fig. S2. Fifty overlapped genes included 13 aforementioned genes. Meanwhile, most of the genes (533) were only among Fury et al. results, whereas 114 genes were only among our results. A small number of overlapping DEGs can be explained by objective variations in the experimental conditions of different studies (type of animals, occlusion time, brain area, bionformatics). But also it can indicate an active spatial–temporal regulation of the gene expression in the brain under conditions of cerebral ischemia. Perhaps, the differences can accumulated as the analyzed area of the brain moves away from the focus of ischemic damage.

The second group of genes was the most numerous (2,672 genes) and included those genes whose expression level changed significantly in the IH but not in the CH after tMCAO. The pattern of transcriptional activity largely reproduced the inflammatory and neurotransmitter-related effects of ischemia in the IH^15,30^. However, these genes reflect the unique effects of ischemia in the brain region containing the area of the ischemic focus rather than far from the focus of damage.

The third group included 34 genes (e.g. *Pla2g3*, *Htra1*, *Tnfrsf11b*, *Iqgap3*, *S100a9*, *Avp*, *Nr4a3*, *Folr1*, and *Pmch*) that are unique to the CH transcriptome reaction. The mRNA levels for these genes changed significantly only in the CH, a finding that reflects effects remote from the area of ischemia. Six of these genes, namely *Parpbp*, *Pmch*, *Th*, *Hcrt*, *P2rx2*, and *S100a9*, exhibited cutoff > 3.5-fold and were identified by GSEA as associated with the p38 signaling pathway mediated by MAPKAP kinases and the signaling pathway for the regulation of TLR by endogenous ligand, which are unique to the CH. This rat tMCAO model reflects ischemia and recovery after stroke in humans. Our study is the first to identify this set of genes in the response to IR injury, and these findings may provide a basis for further biomedical research in this field.

The fourth group of genes is of particular interest. We identified 16 genes (*Hrh3*, *Chst15*, *Drd2*, *Rasd2*, *Drd1*, *Hpca*, *Lrrc10b*, *Slc24a4*, *Scn4b*, *Neu2*, *Gng7*, *Adora2a*, *Asic4*, *Syndig1l*, *Rgs9*, and *Gpr6*) that exhibited opposite changes in expression levels in the two rat brain hemispheres. Using the Functional Annotation Tools PANTHER and DAVID, we found that these genes are associated predominantly with the neurosignaling systems and may be nodes for the regulation or brain neurotransmission after stroke. To examine this regulatory network, we used the signaling pathway depository from the three most representative databases, KP, RP, and WP. Each of these databases has its own specific features and each contains information about the functional annotation of protein gene products and their biological functions^36–38^. However, databases differ in the number of pathways they contain, average number of proteins per pathway, types of biochemical interactions included, and subcategories of pathways they provide. Pathways are also often described at different levels of detail, with different types of data, and with vaguely defined boundaries^39–41^. Therefore, we used a few databases to overcome the subjective features of each. We found that the “Signal transduction” (RP), “Signaling by GPCR” (RP), “Neuroactive ligand-receptor interaction” (KP), “Cocaine addiction” (KP), cAMP signaling pathway (KP), and Dopaminergic synapse (KP) were top neurosignaling pathways that were simultaneously associated with a full set of DEGs in the IH and with the most DEGs from fourth group.

Concomitantly, the top five genes involved in the most signaling pathways were identified as *Gng7* (29 pathways), *Drd1* (eight pathways), *Drd2* (eight pathways), *Adora2a* (seven pathways), and *Rgs9* (six pathways). The role of these genes in ischemia is only partially understood and continues to be actively studied. We have reported downregulation of *Gng7* expression in the rat brain at 24 h after tMCAO^15^. *Gng7* encodes subunit gamma 7 of guanine nucleotide-binding proteins (Gγ7, GNG7). The role of Gγ7 function in dopamine D1 and D2 receptor neurosignaling has been reported^42,43^. Interestingly, D1 receptor-mediated endogenous tissue plasminogen activator upregulation contributes to BBB injury after acute ischemic stroke^44^. Postischemic administration of a D2 receptor agonist reduces cell death by activating mitochondrial pathway following ischemic stroke^45^. D2 and *Drd2* are associated with the regulatory action of neuroactive drugs after stroke^46,47^. Additionally, the role of adenosine A2A receptors, which are encoded by *Adora2a*, under cerebral IR conditions has been reported^48^.

Recently, Ito et al. directly compared the molecular responses between mice that had recovered naturally (spontaneously) from stroke and those that had not recovered, and reported novel molecular signatures that have been masked by studies using external restorative treatments^49^. The RNA-Seq data of Ito et al. revealed a panel of recovery-related genes in the motor cortex of spontaneously recovered mice and highlighted the involvement of the contralesional cortex, particularly the *Adora2a*, *Drd2*, and *Pde10a*-mediated cAMP signaling pathway, in spontaneous recovery^49^. Interestingly, we found overlapping between the gene lists of Ito et al. and our fourth group of genes mentioned above. This overlapping is probably not a coincidence and suggests the functional significance of genetic clustering in the processes of postischemic brain recovery, including using the potential of the CH cells.

A limitation of our study is the lack of different timepoints to provide the dynamic changes of RNA expressions, as well as functional evidence supporting the changes in the mRNA expression of these genes. By establishing the functional interactions between the DEGs, we believe that we have identified changes at the RNA level in both the CH and IH that provide clues about the effects of stroke on the brain.

In conclusion, we comprehensively analyzed changes in the transcriptome in the IH and CH after a focal stroke in a tMCAO rat model. We identified evidence of a bilateral genetic response triggered by ischemic stroke in the cerebral hemispheres at the transcriptome level on a genome-wide scale. The specific genome responses in the CH and IH may be useful for the study of the potential for regeneration of brain cells after stroke.

## Methods

### Animals

White 2-month-old male rats of the Wistar line (weight, 200–250 g) were obtained from the AlCondi, Ltd. animal breeding house (Moscow, Russia) as previously described^31^. All protocols adhered to the Guide for Care and Use of Laboratory Animals to minimize pain and suffering. All methods involving animals are reported in accordance with ARRIVE (Animal Research: Reporting of in Vivo Experiments) guidelines (https://arriveguidelines.org).

### tMCAO model in rats

The tMCAO rat model was applied with magnetic resonance imaging (MRI) as previously described^31^. The rats were decapitated at 24 h after tMCAO (IR group). Rats in the SO group were subjected to a similar surgical procedure under anesthesia comprising neck incision and separation of the bifurcation but without tMCAO. Each experimental group comprised at least five animals. MRI confirmed that all rats in the IR group had a focal hemispheric lesion lying in the subcortex region of the right (ipsilateral) brain hemisphere and that this had spread to the adjacent cortex. MRI showed no pathological changes in the CH of ischemic rats^31^. MRI of ischemic foci after tMCAO is shown in Supplementary Fig. S3. Also, stroke score data (MRI) are shown in Supplementary Table S8.

The subcortical structures of the brain in the IH group (IR-i group) and CH group (IR-c group) of ischemic rats, and in the right (SO-r) and left (SO-l) hemisphere of SO rats were obtained previously^31^. For the study, subcortical structures were taken in the range from + 2 to − 2 mm from the bregma. The samples included the striatum. All IR-i, IR-c, SO-r, and SO-l samples were placed in RNAlater (Ambion, Austin, TX, USA) solution for 24 h at 4 °C, stored at − 70 °C and then used for RNA isolation. RNA integrity was checked using capillary electrophoresis (Experion, BioRad, Hercules, CA, USA). RNA integrity number (RIN) was at least 9.0^31^.

### Real-time reverse transcription polymerase chain reaction (RT–PCR)

cDNA samples obtained earlier were used for RT**–**PCR^31^. The 25-μl PCR mixture contained 2 μl of 0.2 × reverse transcriptase reaction sample, forward and reverse primers (5 pmol each), 5 μl of 5 × reaction mixture (Evrogen Joint Stock Company, Moscow, Russia) including PCR buffer, Taq DNA polymerase, deoxyribonucleoside triphosphates (dNTP), and the intercalating dye SYBR Green I. Primers specific to the genes studied were selected using OLIGO Primer Analysis Software (version 6.31) and were synthesized by the Evrogen Joint Stock Company (see Supplementary Table S2). The amplification of cDNAs was performed using a StepOnePlus Real-Time PCR System (Applied Biosystems, Foster City, CA, USA) in the following mode: stage 1 (denaturation), 95 °C for 10 min; stage 2 (amplification with fluorescence measured), 95 °C for 15 s; 65 °C for 25 s; and 72 °C for 35 s (40 cycles).

### RNA-Seq data

The RNA-Seq data for three pairwise comparisons, IR-i vs. SO-r, IR-i vs. IR-c, and IR-c vs. SO-l, were obtained previously using the Cuffdiff program^31^ and were analyzed further in these experiments. Each of the comparison groups (IR-i, SO-r, IR-c, and SO-l) included three animals. Only genes that exhibited changes in expression of > 1.5-fold and had a *P*-value < 0.05 (*Padj* < 0.05) in the t test adjusted using the Benjamini–Hochberg procedure were analyzed.

### Analysis of real-time RT–PCR data and statistics

Two reference genes *Gapdh* and *Rpl3* were used to normalize the cDNA samples^50^. Calculations were performed using Relative Expression Software Tool (REST) 2005 software (gene-quantification, Freising-Weihenstephan, Bavaria, Germany)^51^. The manual for the site ‘REST.-gene-quantification.info’ was used to evaluate the expression target genes relative to the expression levels of the reference genes. The values were calculated as 2^Ct(ref)–Ct(tar)^, where Ct(tar) is the average threshold cycle (Ct) of the target gene and Ct(ref) is the average Ct of the reference gene. Five animals were included in each comparison group. *P* < 0.05 was considered to indicate significant differences between comparison groups. Additional calculations were performed using Microsoft Excel (Microsoft Office 2010, Microsoft, Redmond, WA, USA).

### Functional analysis

Database for Annotation, Visualization and Integrated Discovery (DAVID) (2021 Update)^52^, Gene Set Enrichment Analysis (GSEA)^53^, and The PANTHER (Protein ANalysis THrough Evolutionary Relationships)^54^ resources were used to annotate the functions of the differentially expressed mRNAs (DEGs). *Padj* < 0.05 was considered to indicate significant differences between comparison groups. Hierarchical cluster analysis of DEGs was performed using Heatmapper (Wishart Research Group, University of Alberta, Ottawa, Canada)^55^. A volcano plot was constructed using Microsoft Excel (Microsoft Office 2010). Violin plots were constructed using BoxPlotR, a web tool for generation of box plots^56^. Cytoscape 3.8.2 software (Institute for Systems Biology, Seattle, WA, USA)^57^ was used to visualize the regulatory network. Additional calculations were performed using Microsoft Excel (Microsoft Office 2010).

### Ethical approval

All manipulations with experimental animals were approved by the Animal Care Committee of the Pirogov Russian National Research Medical University (Approved ID: 15-2015, November 2, 2015) and were carried out in accordance with the Directive 2010/63/EU of the European Parliament and the Council of European Union on the protection of animals used for scientific purposes issued on September 22, 2010.

### Accordance

The methods were carried out in accordance with the relevant guidelines and regulations.

### Informed consent

Informed consent was obtained from all individual participants included in the study.

## Supplementary Information

Supplementary Figure S1.

Supplementary Figure S2.

Supplementary Figure S3.

Supplementary Table S1.

Supplementary Table S2.

Supplementary Table S3.

Supplementary Table S4.

Supplementary Table S5.

Supplementary Table S6.

Supplementary Table S7.

Supplementary Table S8.

## Data Availability

RNA-sequencing data have been deposited in the Sequence Read Archive database under Accession Code PRJNA803984 (SAMN25694602-SAMN25694613, https://dataview.ncbi.nlm.nih.gov/object/PRJNA803984?reviewer=l88f1nds1ng14748kfoujc72r4 (http://www.ncbi.nlm.nih.gov/bioproject/803984)^58^.
