# Supplementary material for: Differential gene expression in the contralateral hemisphere of the rat brain after focal ischemia
Source: Sci Rep. 2023 Jan 11;13:573. doi: 10.1038/s41598-023-27663-8 (PMC9834327; doi:10.1038/s41598-023-27663-8)
Supplement: Supplementary file 1 — Supplementary Figure S1. [file 41598_2023_27663_MOESM1_ESM.docx]

**Differential Gene Expression in the Contralateral Hemisphere of the Rat Brain after Focal Ischemia**

Ivan B. Filippenkov^1^*, Email: filippenkov@img.msk.ru

Julia A. Remizova^1^, Email: utoshkautoshka@gmail.com

Alina E. Denisova^2^, Email: dalina543@gmail.com

Vasily V. Stavchansky^1^, Email: bacbac@yandex.ru

Ksenia D. Golovina^1^, Email: zefeni@yandex.ru

Leonid V. Gubsky^2,3^, Email: [gubskii@mail.ru](mailto:gubskii@mail.ru)

Svetlana A. Limborska^1^, Email: limbor@img.msk.ru

Lyudmila V. Dergunova^1^, Email: lvd@img.msk.ru

^1^Institute of Molecular Genetics of National Research Center “Kurchatov Institute”, Kurchatov Sq. 2, 123182 Moscow, Russia

^2^Department of Neurology, Neurosurgery and Medical Genetics, Pirogov Russian National Research Medical University, Ostrovitianov str. 1, 117997 Moscow, Russia

^3^Federal Center for the Brain and Neurotechnologies, Federal Biomedical Agency, Ostrovitianov str. 1, Building 10, 117997 Moscow, Russia

* Correspondence to: Ivan B. Filippenkov,

E. mail: filippenkov@img.msk.ru, Phone: +7(499)1961858, Fax: +7(499)1960221.

**Supplementary Figure S1**

**Fig. S1.** **Graphical abstract of results.**

**
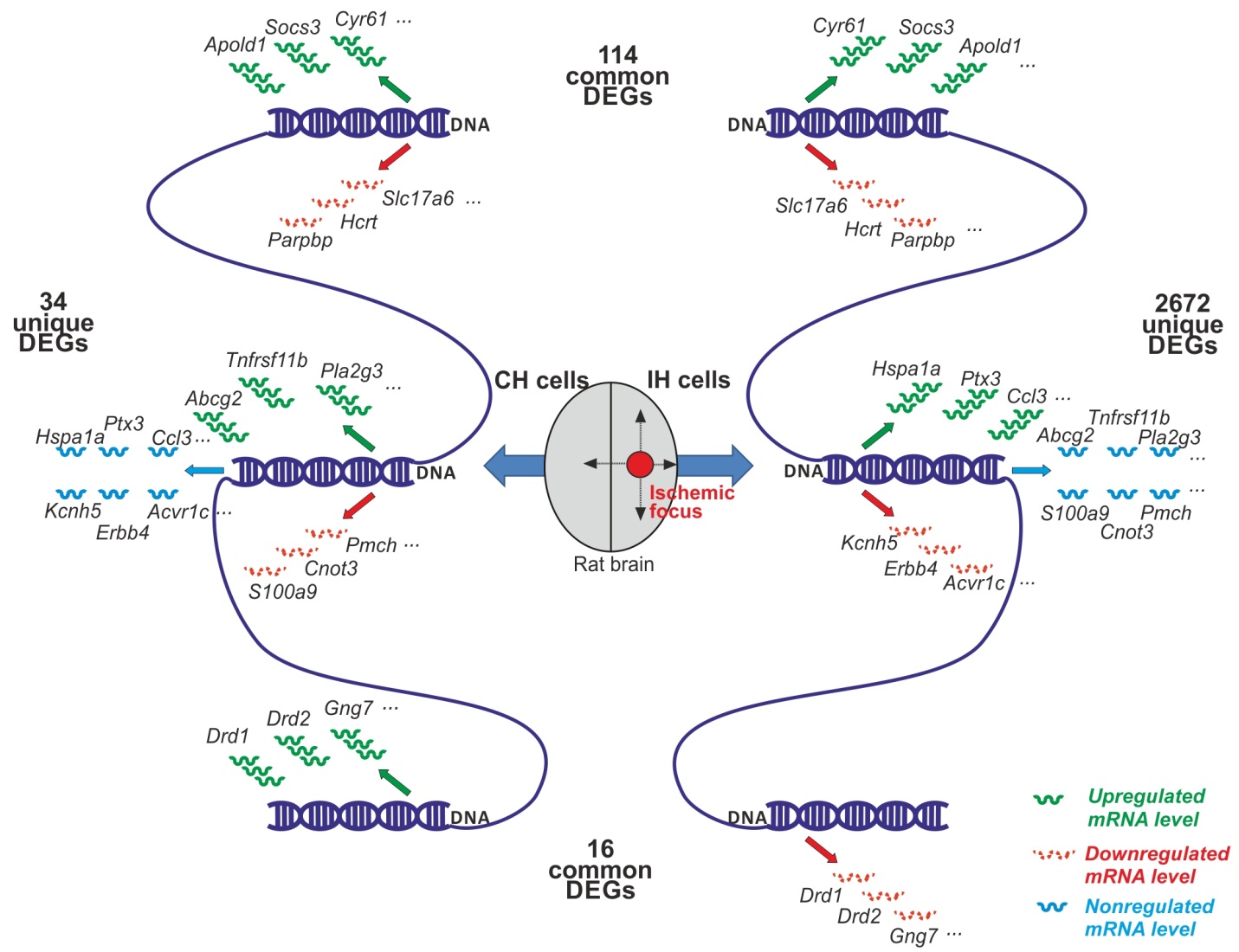
**
